# Antimicrobial Efficacy of Pelargonic Acid Micelles against *Salmonella* varies by Surfactant, Serotype and Stress Response

**DOI:** 10.1038/s41598-020-67223-y

**Published:** 2020-06-24

**Authors:** Govindaraj Dev Kumar, Kevin Mis Solval, Abhinav Mishra, Dumitru Macarisin

**Affiliations:** 1 Center for Food Safety, College of Agricultural and Environmental Sciences, University of Georgia, Griffin, Georgia USA; 2 Department of Food Science & Technology, University of Georgia, Griffin, GA USA; 3 Department of Food Science & Technology, University of Georgia, Athens, GA USA; 4 Office of Regulatory Science, Center for Food Safety and Applied Nutrition, Food and Drug Administration, College Park, MD USA

**Keywords:** Microbiology, Antimicrobials, Applied microbiology, Biofilms, Pathogens

## Abstract

The antimicrobial properties of Pelargonic acid (PA), a component of tomatoes, makes it an attractive candidate as a food additive and sanitizer. The antimicrobial efficacy of PA emulsions generated using surfactants: Tween 80, Triton X100, Sodium Dodecyl Sulfate (SDS) and Quillaja Saponin was evaluated against *Salmonella* serotypes Newport, Oranienburg and Typhimurium. Micelle/dropletsize, and minimal inhibitory concentrations (MIC) were determined. Surfactant type and concentration significantly influenced the antimicrobial efficacy of PA (p < 0.05). Overall, *Salmonella* Newport was the most (p < 0.05) susceptible serotype to PA emulsions. PA emulsions generated with 1.00% SDS had the highest (p < 0.05) antimicrobial activity, with MIC of 7.82 mM against *S*. Newport and 15.62 mM against *S*. Oranienburg/*S*. Typhimurium, respectively. Addition of PA to Trypticase Soy Broth resulted in a decreased growth rate and an increased lag phase duration. Cells exposed to PA formed elongated filaments (>5 µm). Additionally, *Salmonella* serotypes Typhimurium and Newport also formed floccular biofilms. PA emulsions at a concentration of 31.25 mM generated using 1% SDS and 1% Quillaja saponin resulted in >6 log CFU/ml reduction in *Salmonella *population. Althought all PA emulsions evalauted inhibited *Salmonella*, morphological changes to this antimicrobial varied substantially among the *Salmonella* serotypes tested.

## Introduction

Non-typhoidal *Salmonella* are one of the most commonly implicated bacterial agents of foodborne infection in the United States^1^. A wide variety of *Salmonella* serotypes have been responsible for outbreaks associated with poultry, produce, beef, low moisture food and petfood^2^. The heterogeneity among food matrices indicates that different levels of stress tolerances could occur among *Salmonella* serotypes^3^. Further, the rise of Anti-Microbial Resistance (AMR) in *Salmonella* is threat of immediate concern with dire ramifications that requires the exploration of antimicrobial agents and a better understanding of antimicrobial resistance strategies in *Salmonella*^4^.

Antibiotic resistant bacteria result in 700,000 deaths every year globally and have been on the rise^5,6^. Sanitizers, such as quarternary ammonium compounds (QACs), when used at sub-lethal concentrations, can contribute to the development of antibiotic resistance in bacterial pathogens^7^. There is a strong evidence that antibiotic resistance and biocide resistance in bacteria are linked; the phenomenon is referred to as biocide-antibiotic cross-resistance or cross-tolerance^8^. Thus, the possibility that human enteric pathogens could develop resistance against conventional biocides along with multidrug-resistance, calls for the development of new sanitizers and antimicrobial compounds.

Plants have been used for millennia and remain a promising source of antimicrobial compounds to substitute for currently used sanitizers and antibiotics. Plant derived fatty acids such as lauric acid, oleic acid, palmitoleic acid, caproic, caprylic, and capric acid have demonstrated antimicrobial activity against both Gram-positive and Gram-negative foodborne bacterial pathogens^9,10^. The antimicrobial activities of certain fatty acids result from their ability to disrupt bacterial membrane lipids, alter membrane fluidity or form hydroperoxides leading to oxidative damage^11^. Pelargonic acid (PA) is a commonly used antifungal agent with a Generally Recognized as Safe (GRAS) status and is a component of the tomato exometabolome. Recently the antimicrobial activity of PA against *Salmonella* was demonstrated^11^ indicating that the fatty acid had better antimicrobial activity when dispersed as a micelle using Quillaja saponin than its unmodified form, hence highlighting its potential as a GRAS antimicrobial food additive or as a sanitizer.

Though a variety of fatty acids demonstrate antimicrobial activity, their efficacy is often diminished by their poor solubility in water and phase separation in an aqueous medium^11^. Emulsification of fatty acids can increase their miscibility and dispersion in water^12^, resulting in improved antimicrobial activity^11,13^. Surfactants such as Tween 80, SDS and Quillaja saponin, a triterpene glycoside from the bark of the *Quillaja saponaria* Molina can be used to produce emulsions of fatty acids and essential oils that have antimicrobial properties. Surfactants can influence the antimicrobial efficacy of an emulsion due to their charges and their placement on the colloidal particle, thereby influencing the particle size and the amount of antimicrobial compound that is in contact with the target bacterial cell^14–17^.

The surfactant choice and concentration can either, synergistically strengthen or impede the antimicrobial activity of an emulsion^16,18^. Previous research indicated that exposure to sublethal concentration (20 mM) of PA induced a transitional morphological change (filament formation) in *Salmonella*^11,19,20^. Formation of diverse phenotypes by bacteria in challenging environments is a strategy to overcome antimicrobial hurdles^21,22^. Determining morphological changes in *Salmonella* during the exposure to PA emulsions formed using different surfactants could provide a better understanding of the antimicrobial’s mechanistic action of and means of adaptation by the pathogen.

The minimal inhibitory concentrations (MIC) of aqueous emulsions of PA generated using Tween 80, Triton X100, SDS and Quillaja saponin were determined against *Salmonella* serotypes Newport isolated from cattle, Oranienburg isolated from nut and a reference Typhimurium strain for antimicrobial testing. Bactericidal efficacy of the most potent emulsions was evaluated against a cocktail of the *Salmonella* serotypes after treatment durations of 30 s and 5 min. Growth kinetics and cell physiology of the *Salmonella* serotypes were tested in presence of inhibitory PA emulsions to study adaptative mechanisms to the antimicrobial. The results of this study are intended to bridge the current knowledge gap on the role of surfactant type in antimicrobial efficacy of PA to aid in the development of fatty acid-based washes and rinses for the food industry.

## Materials and Methods

### Micelle and droplet size measurements

Droplet size distribution and polydispersity (intensity-based size distribution) measurements of emulsions were conducted at 20 °C by Dynamic Light Scattering that examines the fluctuations in light scattering due to brownian motion of the emulsion droplets, using a Zetasizer Nano ZS device (Malvern, Southbough, MA). Briefly, 5 ml of emulsion samples were diluted in 50 ml of deionized water. Then, 10 ml of the diluted samples were filtered through a 0.40 µm nylon filter (Syringe-filters, Cole Parmer, UK) and vortexed. Mean hydrodynamic diameter of emulsion droplets was calculated via cumulative analysis (z-average) and the size distributions were defined by the polydispersity index (PdI) and the size distribution graph.

### Bacterial Culture

*Salmonella enterica* subsp. *enterica* serotype Newport (11590 K, beef isolate, GFP labelled), *S*. Oranienburg (1839, pecan isolate) and *S*. Typhimurium (CDC 6516-60/ATCC 14028) were obtained from the Center for Food Safety, University of Georgia, Culture Collection. Cultures were revived by two successive 24 h transfers in 10 ml Tryptic Soy Broth (TSB, Acumedia Neogen Corporation. Lansing, MI) with antibiotics (100 µg/ml Ampicillin for *S*. Newport and Typhimurium and 50 µg/ml Nalidixic acid for *S*. Oranienburg), at 35 °C. Then, the cultures were streaked on Tryptic Soy Agar (TSA, Acumedia, Neogen Corporation. Lansing, MI) with antibiotics and incubated at 35 °C for 24 h. Individual colonies were confirmed serologically using a latex agglutination (Oxoid, Ogdensberg, NY). Isolated colonies were then streaked on XLT4 (Xylose Lysine Deoxycholate agar with tergitol 4, Acumedia, Neogen Corporation, Lansing, MI) at 35 °C for 24 h and observed for typical black colonies. Stock cultures were prepared by streaking a colony of each *Salmonella* serotype on TSA with antibiotics and incubating the plate at 35 °C for 24 h. The colonies were then scraped with a sterile loop and suspended in Phosphate Buffered Saline (PBS) and adjusted to a population of ca. 6 Log CFU/ml.

### Emulsion preparation

A 1 M stock emulsion of n-Pelargonic acid, 97% (Pelargonic acid, Acros Organics, New Jersey, USA) in water was prepared using TritonX-100, (ICN Biomedicals, Inc., Aurora, OH), Tween 80 (Fisher Chemical, Fair Lawn, NJ), Sodium Dodecyl Sulfide (SDS, Fisher Chemical), Sodium lauroyl sarcosinate (Sarkosyl, Fisher Chemical) and saponin from quillaja bark (Sigma-Aldrich, St. Louis, MO) at concentrations of 1%, 0.1% and 0.01% (w/v). Stock emulsions (1 M) were prepared by adding 1.58 g of PA to 10 ml of water with 1%, 0.1%, and 0.01% (w/v) of surfactant. The mixture was agitated on a vortexer (Vortex-2 Genie, Scientific Industries, Bohemia, NY, USA) at maximum speed setting of 10 (3200 rpm) for 1 min to form the emulsion.

### MIC determination

Minimal inhibitory concentration of the emulsions was determined by the modified 96 well plate Resazurin assay^23^. Briefly, stock solutions of the PA emulsions were serially diluted into Iso-sensitest broth (Oxoid Ltd., Hants, UK) containing Resazurin (Biotium, Hayward, CA) (0.001 g/ml) and inoculated with ca. 5 Log CFU/ml of *Salmonella*. Serial dilutions were performed to obtain PA concentrations ranging from 500 mM to 2.73 mM of each emulsion. The 96 well plates were incubated for 24 h at 37 °C and the wells containing the serial dilutions of the emulsions were observed for a change in color from blue to pink indicating bacterial growth. The lowest dilution at which no bacterial growth was determined (blue well) was considered the minimum inhibitory concentration. The MICs of the individual surfactants at concentrations of 1%, 0.1% and 0.01% (w/v) and PA without surfactant that was suspended in water by vortexing for 1 min were determined as comparative controls using the modified 96 well plate Resazurin assay. Each test was performed three times and an average of the three MIC values were used.

### Bactericidal activity

Concentration of PA emulsions that were inhibitory were evaluated for their bactericidal activity against *Salmonella* serotypes. After determining the MIC as described in the previous section, wells containing the lowest inhibitory concetration of the pelargonic acid emulsion were neutralized using Dey-Engley neutralizing broth (Neogen Corp., Lansing, MI) and enumerated on TSA by spread plating with appropriate dilutions. Emulsions that resulted in a reduction of bacterial populations ≥3 log CFU/ml were considered bactericidal^24^. Each experiment was performed three times and an average of the three MIC values were used.

### Antimicrobial efficacy against *Salmonella* cocktail

A PBS suspension consisting of a cocktail of *Salmonella* serotypes Newport, Typhimurium and Oranienburg at a concentration of 9 Log CFU/ml was exposed to 31.25 mM and 15.62 mM of PA-Quillaja Saponin (1%) and PA – SDS (1%). The concentrations of emulsions selected were inhibitory to all three serotypes of *Salmonella*. Exposure durations tested were for 30 s and 5 min. Following exposure, the fatty acid was neutralized using Dey-Engley neutralizing broth and enumerated by spread plating after appropriate diltuons. Each experiment was performed three times.

### Growth Kinetics of *Salmonella* in presence of PA emulsion

*Salmonella* serotypes Newport, Typhimurium and Oranienburg were grown in presence of TSB at 37 °C with 15.6 mM of PA emulsion generated using 1% Quillaja saponin and TSB with 7.80 mM of PA emulsion generated using 1% SDS. The concentration of the emulsion used was inhibitory to *S*. Newport (p < 0.05) and was sub-inhibitory to *S*. Typhimurium and *S*. Oranienburg. Initial population of 5 log CFU/ml of *Salmonella* serotypes were grown in 50 ml centrifuge tubes **(**VWR International, Radnor, PA) of TSB at 37 °C. The cells were neutralized using Dey-Engley neutralizing broth and enumeratd at 0, 2, 4, 8, 12, 16, 20 and 24 h by droplet plate method on TSA with appropriate serial dilutions. Controls for the experiment were grown without the presence of PA emulsions with identical culture conditions. Growth curves were plotted and analyzed using MATLAB (The Math Works, Natick, MA, Version 2017). Baranyi model^25^ was used as the primary model to predict the bacterial population as a function of time.

### Assessment of morphology

Cell morphology of *Salmonella* serotypes grown in TSB amended with 15.62 mM of PA emulsion generated using 1% Quillaja saponin and TSB with 7.80 mM of PA emulsion generated using 1% SDS at, 37 °C after 24 h of growth were evaluated through phase contrast microscopy (Nikon Eclipse Ci-L; Nikon Corp., Japan). Wet mount preparations of 20 µl and studied using a Nikon 100X Plan Apo objective (Nikon Eclipse 50i; Nikon Corp., Japan) with the Nikon Elements software through with a scale of 5 µm to differentiate and enumerate cells that were larger than 5 µm (filaments, F) and cells that were smaller than 5 µm (regular, R). and aggregates of cells (>5 cells) were considered as floccules. Direct counts of filamentous cells and regular-sized cells were determined from 3 experimental replicates (3 fields per slide) for each treatment and time point. The average counts of filamentous cells (F) and regular cells (R) were used to determine the F:R ratio. Each field had between 50 to 200 cells.

The structure of the floccular biofilms was studied using Confocal Laser Scanning Miroscopy (CLSM). Floccular biofilm was further confirmed by staining with FilmTracer calcein red-orange (Molecular Probes, Eugene, OR) biofilm stain. Speciment staining with FilmTracer calcein red-orange was conducted according to manufacturer’s instructions. After an hour of incubation at room temperature in the dark, the sample was observed using a  Zeiss 880 laser scanning  confocal microscope with a 63×/1.4 NA oil immersion Plan Apochromatic objective at 576/590 nm.

### Statistical analysis

The experiments for this study were designed using a randomized blocked design factorial treatment arrangement, repeated measures with sampling, blocked on replication (Table 1). The statistical analysis of the data was performed with analysis of variance (ANOVA) using JMP Pro 14 (SAS Institute, Cary, NC). Tukey-Kramer honestly significant difference test was used to determine differences between mean values of the MIC and droplet size of different tests at a 95% confidence level.

**Table 1:** Characteristics of PA emulsions generated using 0.01, 0.1 and 1% surfactants.

Emulsion	Concentration % (w/v)	pH	Z-average diameter(nm)	Polydispersity index (PdI)
SDS	1	3.78	3220.50 ± 133.50_**a**_	1
	0.1	3.43	1499.67 ± 112.90_**b**_	0.92
	0.01	3.45	289.70 ± 27.44_**c**_	0.36
Quillaja saponin	1	3.59	153.30 ± 1.82_**a**_	0.24
	0.1	3.79	169.20 ± 17.22_**ab**_	0.31
	0.01	3.57	169.30 ± 2.09_**b**_	0.23
Triton X100	1	3.52	112.10 ± 3.50_**a**_	0.22
	0.1	3.52	114.03 ± 6.29_**a**_	0.42
	0.01	3.55	461.55 ± 38.25_**b**_	0.38
Tween 80	1	3.86	79.51 ± 0.26_**a**_	0.10
	0.1	3.25	76.15 ± 0.31_**b**_	0.04
	0.01	3.33	86.76 ± 1.31_**c**_	0.19

Z-average diameter values that are preceded by a different letter are significantly (p < 0.05) different from each other for the same surfactant.

To evaluate the correlation between antimicrobial efficacy of PA and average micellar/droplet diameter of PA emulsions, the ordinary least squares regression analysis was applied to the MIC values and the corresponding average micellar/droplet diameter.

## Results

### Micelle characteristics

The micelle size of PA emulsions varied depending on the type and concentration of surfactant. In PA emulsions generated using SDS, micelle diameter was positively associated with surfactant concentration, specifically 1.00% SDS emulsion had the largest (3220.50 ± 133.50 nm) micelles, significantly (p < 0.05) smaller micelles (1499.67 ± 112.90 nm) in emulsions produced with 0.10% SDS, and those generated with 0.01% SDS had the smallest micelles diameters (289.70 ± 27.44 nm) (Table 1). In contrast, in all other surfactants tested, no positive association between micelle diameter and surfactant concentration was observed. Nevertheless, the diameter of micelles varied significantly (p < 0.05) with surfactant concentration, except for Quillaja saponin and TritonX 100 at 1.00 and 0.10% (Table 1). The antimicrobial activity of PA emulsions generated using Quillaja saponin against all three *Salmonella* strains indicated that a positive correlation existed between higher micellar size and inhibitory activity. This correlation was highest for *S*. Newport as compared to other two serotypes. For Tween treatment, there was very weak correlation for *S*. Newport and *S*. Oranienburg, whereas the ‘r’ value was 0.38 for *S*. Typhimurium. For Triton, the correlation coefficient ranged between −0.25 and 0.10, whereas for SDS, the correlation coefficient was strongly negative for *S*. Newport (−0.86) (Fig. 2). The correlation coefficient could not be calculated for the other two strains as the MIC values were constant for all the experimental micellar sizes.

The average pH of 1 M stock solutions of the pelargonic acid emulsions generated using the various surfactants was 3.55 ± 0.18. Polydispersity index varied with both, the type and concentration of the surfactant. The positive association between the value of the polydispersity index and surfactant concentration, was observed only for SDS (Table 1).

### Inhibitory activity of PA emulsions against *Salmonella*

**Figure 1:**
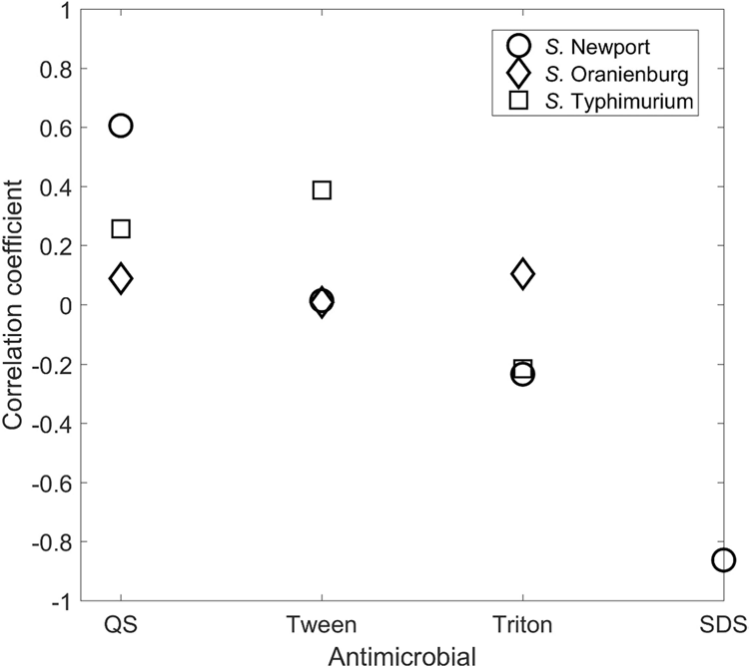
Correlation of average micelle/droplets diameter estimates (*x*) vs. antimicrobial efficacy [value invers to MIC] (*y*) determined for all three *Salmonella* serotypes. The black line is the result from the ordinary least squares regression analysis, i.e. *y* = − 0.009(*x*) + 35.222 and R = 0.409. The red dotted lines are the 95% prediction interval from the regression.

The MIC of PA against all three *Salmonella* serotypes was 125 ± 0.00 mM. Quillaja Saponin did not inhibit *Salmonella* growth at 1%, 0.1% and 0.01%. When emulsions of PA were formed using Quillaja saponin, the antimicrobial activity, as denoted by a decrease in MIC, increased significantly (p < 0.05). Of the three serotypes of *Salmonella* tested, *S*. Newport was most susceptible to PA (p < 0.05). The lowest concentration of PA emulsions that was inhibitory against *S*. Newport was 7.82 ± 0.00 mM and was produced using 1% SDS (p < 0.05). The MIC of PA emulsion produced using 1% SDS for *S*. Oranienburg and *S*. Typhimurium, was 15.62 ± 0.00 mM (Fig. 1). No significant difference in the MIC of PA emulsions produced using 1%, 0.1% and 0.01% SDS was observed against *S*. Oranienburg and *S*. Typhimurium (Fig. 1).

**Figure 2:**
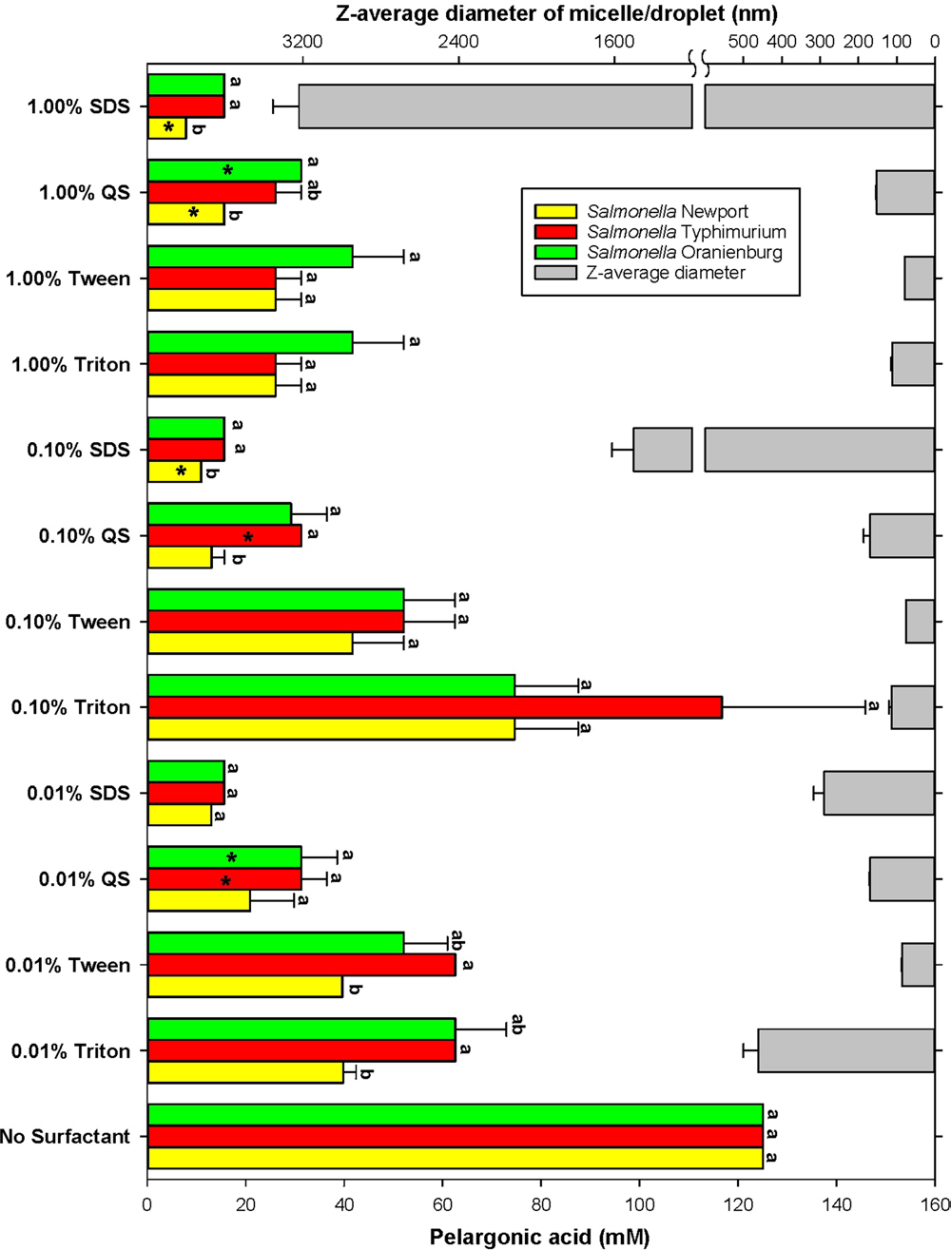
MIC of pelargonic acid determined for *Salmonella* serotypes Newport, Oranienburg and Typhimurium in function of surfactant type and concentration. Colored bars (left *y* axis) present average (n = 3) MIC values ± SE. MIC values that are designated by a different letter are significantly (p < 0.05) different from each other among *Salmonella* serotypes within the same treatment (surfactant and its concentration). MIC values marked by the asterisk were also bactericidal. Grey bars (right *y* axis) are average micelle/droplet diameters (n = 3) ± SE of PA emulsions generated using 0.01, 0.1 and 1% of either: SDS, Quillaja Saponin (QS), Triton X100 (Triton), Tween 80 or (Tween).

Similar to SDS-based emulsions, PA emulsions generated using 1% and 0.1% Quillaja saponin were significantly more inhibitory to *S*. Newport with an MIC of 15.62 ± 0.00 mM and 13.02 ± 4.51 mM respectively (p < 0.05) (Fig. 1). Against serotypes Oranienburg and Typhimurium, PA emulsions generated with 1% and 0.1% Quillaja saponin had an average MIC of 29.42 ± 6.98 mM.

No significant differences in susceptibility was observed among the *Salmonella* serotypes when treated with 0.01% Quillaja saponin generated PA emulsions (MIC of 27.77 ± 6.88 mM) (Fig. 1).

Non-ionic surfactants, Tween 80 or Triton X 100 were not as effective as SDS and Quillaja saponin in improving the antimicrobial activity of PA. Emulsions of PA generated using 1.00% Tween 80 or 1.00% Triton X 100 were inhibitory to the *Salmonella* serotypes tested at 31.25 ± 9.02 mM (Fig. 1). PA emulsions generated using 0.1% Tween 80 had an average MIC of 48.61 ± 16.47 mM against the *Salmonella* serotypes tested. MIC of PA emulsions generated using 0.1% SDS and Quillaja saponin were significantly (p < 0.05) more efficacious against *Salmonella* than those of Tween 80 and Triton X100 (39.62 ± 15.37 mM) (Fig. 1). *S*. Newport was significantly more susceptible to PA emulsions generated using 0.01% Tween 80 (39.79 ± 15.51 mM) and TritonX 100 (39.62 ± 15.37 mM) than other serotypes (p<0.05). The average MIC of PA emulsions generated using 0.01% Tween 80 and Triton X100 against *S*. Oranienburg and Typhimurium was 57.29 ± 12.16 mM.

### Bactericidal activity of emulsions

Suspensions generated using Triton X100 and Tween 80 were not bactericidal at their MIC. Bactericidal activity was observed for suspensions of PA using 0.1% (10.93 ± 00 mM) and 1% SDS (7.82 ± 00 mM) respectively, against *S*. Newport (Fig. 1). Quillaja saponin (0.01%) generated PA suspensions were bactericidal to *S*. Typhimurium and *S*. Oranienburg at a concentration of 31.25 ± 00 mM (Fig. 1). Quillaja saponin 1%, generated PA suspensions were bactericidal to *S*. Newport and *S*. Oranienburg at 15.62 ± 00 mM and 31.25 ± 00 mM respectively. Emulsion generated using 0.1% Quillaja saponin was bactericidal at a concentration of 31.25 ± 00 mM against *S*. Typhimurium, indicating that serotype and surfactant-based variation significantly affected bactericidal activity of the emulsions (p < 0.0001).

### Growth kinetics of *Salmonella*

*Salmonella* serotypes Newport, Oranienburg and Typhimurium were grown in TSB containing PA emulsions generated using Quillaja saponin and SDS. The emulsions were used at a concentration that was inhibitory to *S*. Newport, 5.62 mM, PA with 1% Quillaja saponin and 7.82 mM PA with 1% SDS. Growth rates and physiological adapatations to PA were compared among the three serotypes of *Salmonella* to understand the effects of PA on bacterial growth and the physiological adaptations undergone by the organisms to overcome PA associated challenge.

**Figure 3:**
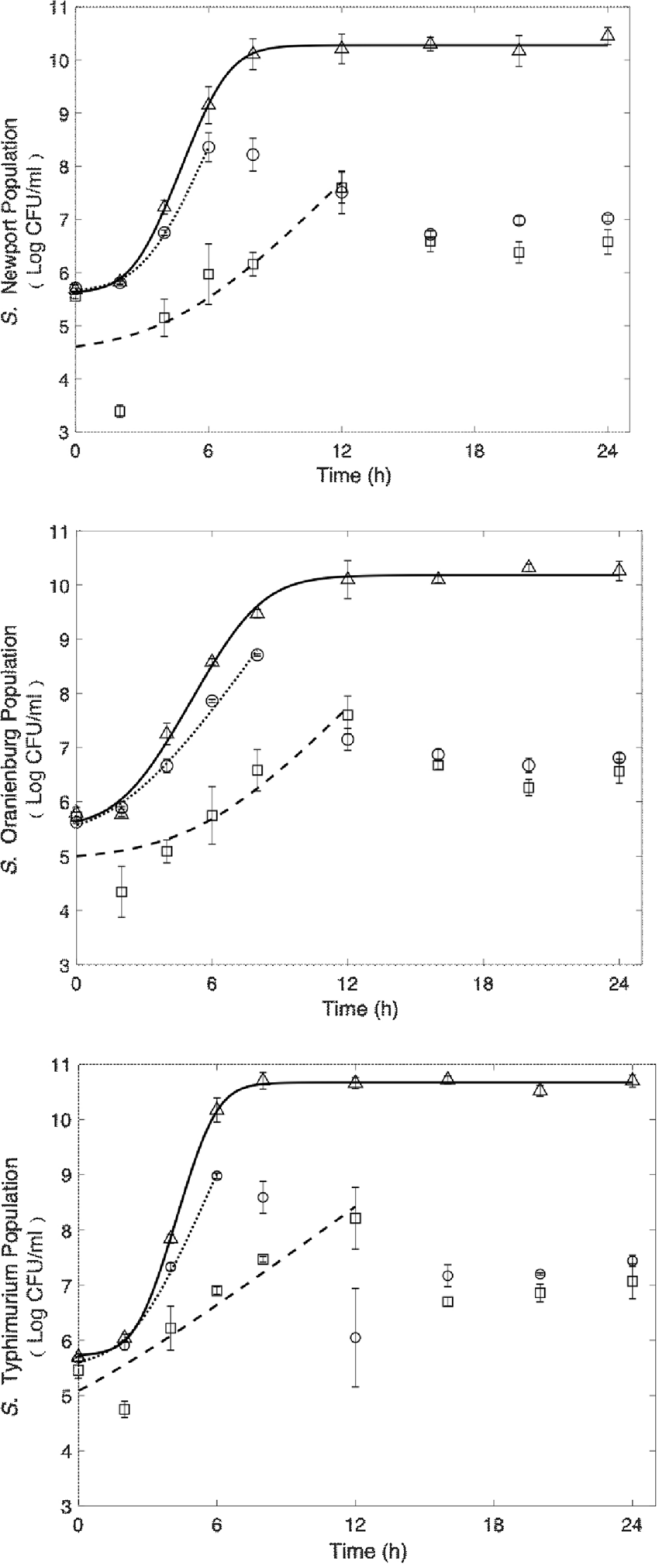
Fitted Baranyi models for the growth data of three *Salmonella* strains at 37 °C. Solid lines and triangular markers (Controls: no antimicrobial; 100 µl 7-log inoculum + 9.9 ml TSB); Dotted lines and circular markers (100 µl 7-log inoculum + 9.9 ml TSB + 156.25 µl 1 M PA with 1% saponin); Dashed lines and square markers (100 µl 7-log inoculum + 9.9 ml TSB + 78.25 µl SDS-PA).

Figure 3 shows the fitted Baranyi models (Baranyi and Roberts, 1994) for *Salmonella* serotypes Newport, Oranienburg, Typhimurium, grown in TSB (control) and TSB amended with 15.62 mM PA with 1% Quillaja saponin and 7.82 mM PA with 1% SDS. While the control samples demonstrated a full growth curve with the maximum population density of 10.57 log CFU/ml, none of the PA treated samples could reach the maximum population density after the initial growth. As such, a full Baranyi model was fitted to the control sample growth data, whereas a reduced Baranyi model was fitted to the growth data showing a growth pattern in both the treated samples. As it is evident from the growth curves, the lag phase duration was the minimum for control samples for all three strains (2.90, 2.72, and 2.54 h for *S*. Newport, *S*. Typhimurium, and *S*. Oranienburg, respectively). For the same strains and for samples treated with SDS and pelargonic acid, the lag phase durations were calculated as 3.36, 2.53, and 5.84 h, respectively. The maximum growth rates (log CFU/h) for these treatments were estimated as 0.99, 0.97, and 0.58, as compared with the control sample growth rates of 1.26, 1.62, and 0.87 log CFU/h for *S*. Newport, *S*. Typhimurium, and *S*. Oranienburg, respectively. These values suggest that although lag phase duration was not affected much by SDS generated PA emulsion, the maximum growth rate was reduced considerably. The samples treated with Quillaja Saponin generated PA emulsions were further impacted, as reflected by the lag phase durations of 4.84, 1.09, and 5.84 h, and maximum growth rates of 0.42, 0.30, and 0.43 log CFU/h for *S*. Newport, *S*. Typhimurium, and *S*. Oranienburg, respectively. All three strains had a considerable drop of population within 12-hours of treatment with either PA emulsion.

### Morphological diversity

Filament formation was significantly influenced by the type of *Salmonella* serotype (p < 0.05) exposed to PA emulsions with *S*. Oranienburg forming the significantly higher number of filaments (p < 0.05) (Table 2). Exposure of *S*. Oranienburg cells to 7.80 mM of PA emulsion generated using 1% SDS for 24 h resulted in the highest number of filament formation with an F:R of 2.18 ± 1.53 (p < 0.05). *S*. Typhimurium formed the lowest number of filaments after exposure to the PA emulsions generated using both surfactants (p < 0.05) with F:R of 0.24 ± 0.18 and 0.07 ± 0.03 for Quillaja saponin and SDS generated PA emulsions respectively (Table 2).

**Table 2:** Differences in the ratio of regular cells:filaments and floccule formation by *S*. Oranienburg, *S*. Newport and *S*. Typhimurium in response to PA (7.80 mM) +1% SDS emulsions and PA(15.62 mM) +1% Quillaja saponin. Treatments resulting significantly different ratios of filaments to regular cells (F:R) and floccule formation (p < 0.5) have been indicated by different alphabet.

*Salmonella* Serotype	PA emulsion	F:R	Floccules
**Oranienburg**	7.80 mM PA emulsion + 1% SDS	0.53 ± 0.23_ab_	0_c_
**Oranienburg**	15.62 mM PA + 1% Quillaja saponin	2.18 ± 1.53_a_	0_c_
**Typhimurium**	7.80 mM PA emulsion + 1% SDS	0.07 ± 0.03_b_	18.3 ± 1.52_a_
**Typhimurium**	15.62 mM PA + 1% Quillaja saponin	0.24 ± 0.18_b_	8.33 ± 6.65_b_
**Newport**	7.80 mM PA emulsion + 1% SDS	0.63 ± 0.16_ab_	2.33 ± 0.57_bc_
**Newport**	15.62 mM PA + 1% Quillaja saponin	0.32 ± 0.08_b_	2.66 ± 0.57_bc_

Floccule formation was significantly influenced by both the serotype (p < 0.0001) of *Salmonella* and the PA emulsions (p < 0.05) the cells were exposed to. Exposure of *S*. Typhimurium to 7.80 mM of PA emulsion generated using 1% SDS resulted in significantly higher floccules than all other treatments (p < 0.05) that resulted in floccule formation. *S*. Oranienburg did not form floccules upon exposure to both SDS and Quillaja saponin generated PA emulsions (Table 2, Fig. 7).

**Figure 4:**
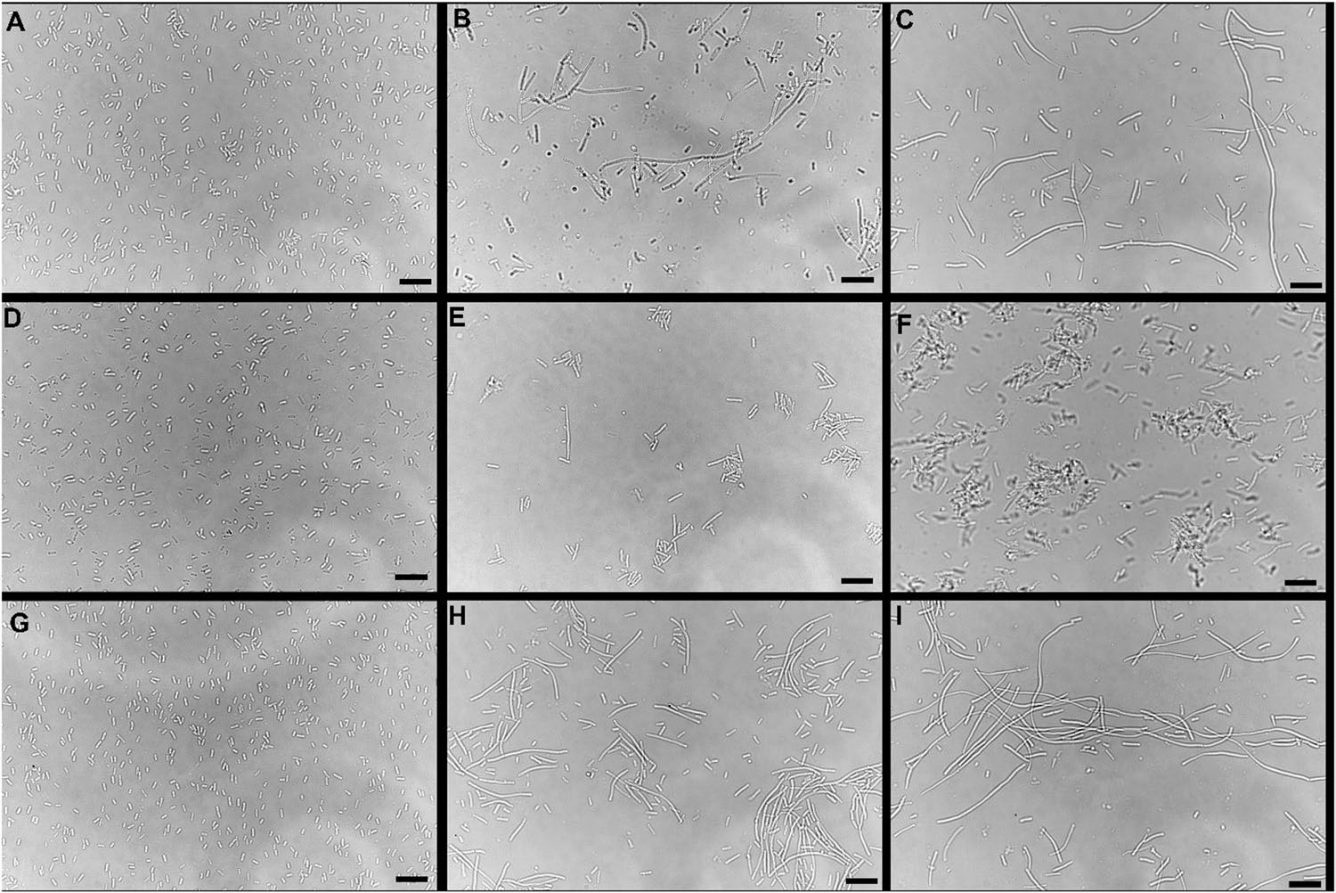
Phase contrast micrographs of (**A**) *Salmonella* Newport cells grown for 24 h in TSB, (**B**) TSB with 7.81 mM PA emulsions generated using 1% SDS, (**C**) TSB with 15.62 mM PA emulsions generated using 1% Quillaja Saponin. (**D**) *Salmonella* Typhimurium cells grown for 24 h in TSB, (**E**) TSB with 7.81 mM PA emulsions generated using 1% SDS, (**F**) TSB with 15.62 mM PA emulsions generated using 1% Quillaja Saponin. (**G**) *Salmonella* Oranienburg cells grown for 24 h in TSB, (**H**) TSB with 7.81 mM PA emulsions generated using 1% SDS, (I) TSB with 15.62 mM PA emulsions generated using 1% Quillaja saponin. Bars at the bottom right corners represents 10 µm in length.

**Figure 5:**
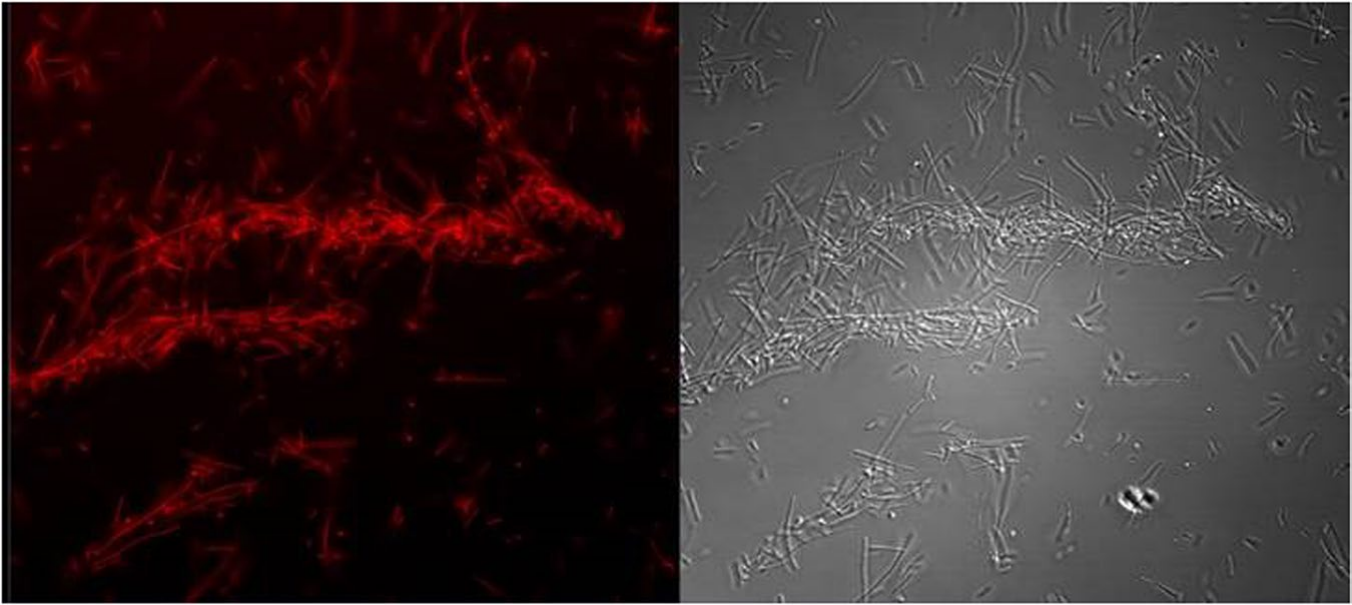
Confocal micrograph of floccular biofilms containing both regular cells and filaments of *S*. Newport. Areas stained in red indicate the presence of EPS.

**Figure 6:**
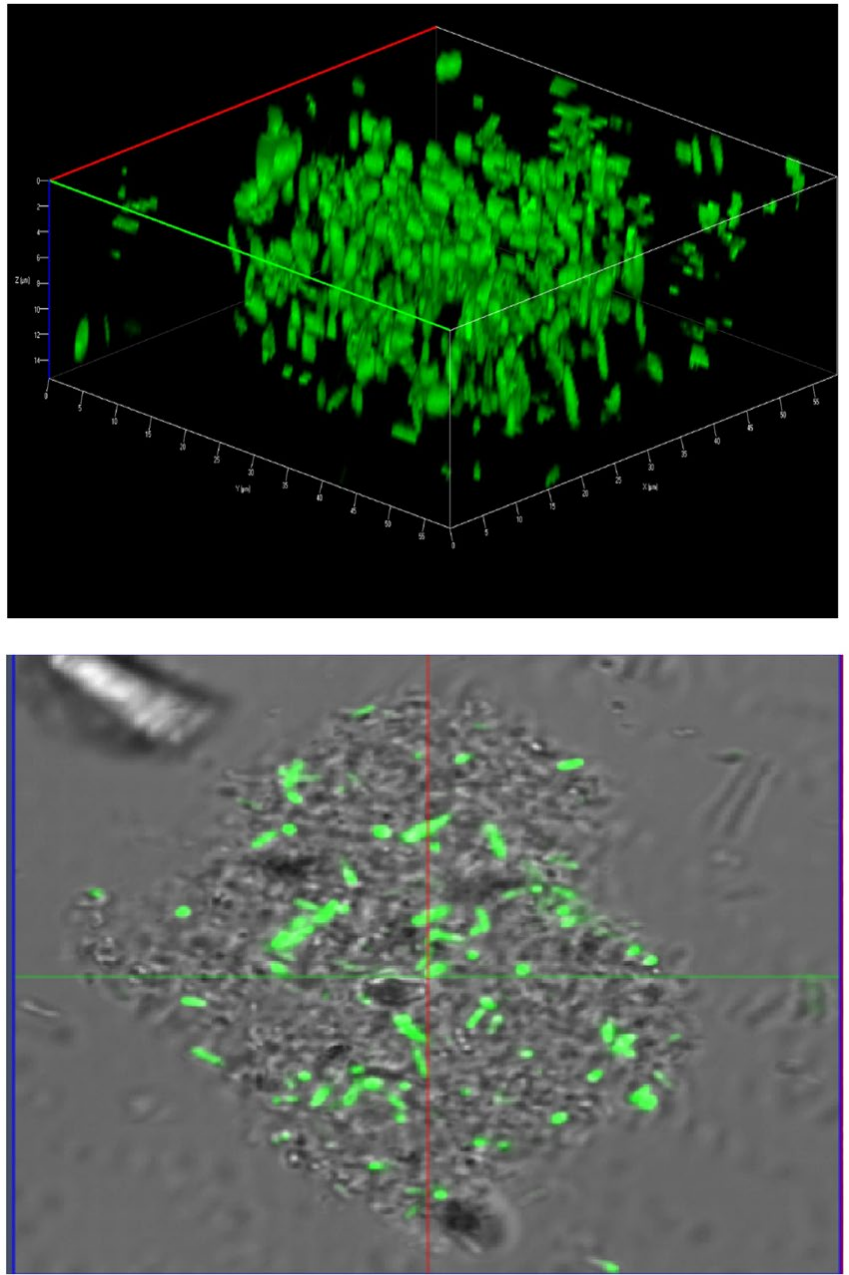
Confocal micrograph demonstrating the architecture of *S*. Typhimurium cells forming floccular biofilms.

**Figure 7:**
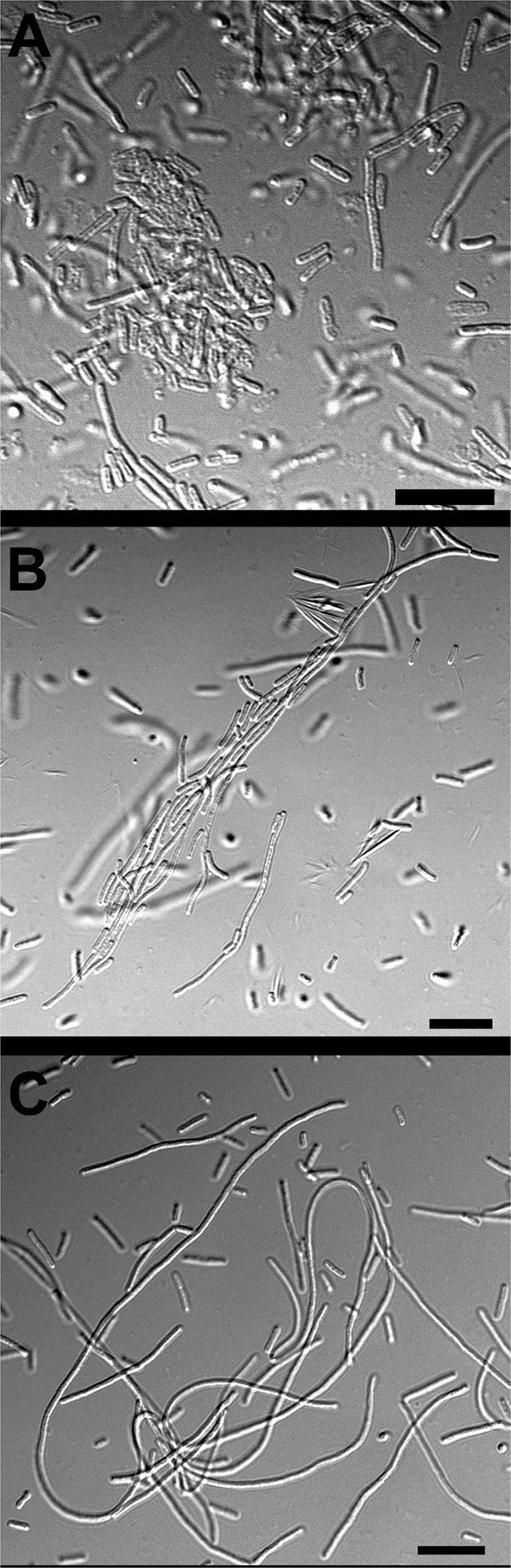
Confocal micrograph demonstrating differences in (**A**) *S*. Newport (**B**) *S*. Typhimurium and (**C**) *S*. Oranienburg response to PA emulsions. Bars at the bottom right corners represents 10 µm in length.

Microscopy analysis of PA-challenged *S*. Typhimurium cells after 24 h growth in TSB indicated that the cells formed aggregates and were encased in a film of exopolymeric substance (Fig. 4). Observation using confocal laser scanning microscopy using optical Z sectioning demonstrated that cellular aggregates (herein reffered as floccules) had a thickness of 10–20 µm and consisted of *Salmonella* cells and EPS (Figs. 4-panel E and F, 5, 6). CLSM micrographs of the stained floccules further confirmed that both filaments and regular size cells were constitutents of these floccular biofilms (Figs. 5, 7).

*Salmonella* Newport was the most susceptible serotype to the concentration of PA used in TSB. In response to both Quillaja saponin- and SDS-generated emulsions, *S*. Newport formed filaments (> 5 µm) and regular sized cells (Figs. 4, 7). The filaments formed by *S*. Newport were thicker than filaments formed by other serotypes with certain filaments displaying cellular dysplasia indicating structural damage to certain filaments (Fig. 4, panel b, c).

### Antimicrobial efficacy of PA emulsions against a *Salmonella* cocktail

**Figure 8:**
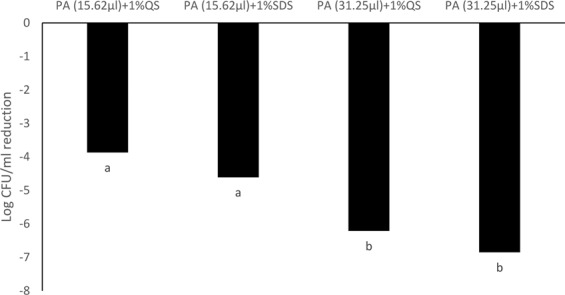
Antimicrobial efficacy of PA-SDS (1%) and PA-Quillaja saponin (1%) emulsions at concentrations of 31.25 and 15.62 mM against a *Salmonella* cocktail consisting of *S*. Newport, *S*. Typhimurium and *S*. Oranienburg after 30 s. Treatments resulting reductions of *Salmonella* cocktail that are significantly different (p < 0.5) have been indicated by different alphabet.

Emulsions containing 31.25 mM PA generated using 1% SDS was most effective against the *Salmonella* cocktail after an exposure duration of 30 s, resulting in a 4.7 log CFU/ml reduction (p < 0.05). Quillaja saponin (1%) based PA emulsions (31.2 mM) had significantly lower antimicrobial efficacy and reduced *Salmonella* by 2.15 log CFU/ml (p < 0.05) (Fig. 8). No significant differences were observed in antimicrobial activity between PA emulsions generated using 1% SDS (1.62 log CFU/ml) or 1% Quillaja saponin (0.77 log CFU/ml) when used at a concentration of 15.62 mM (Fig. 8).

**Figure 9:**
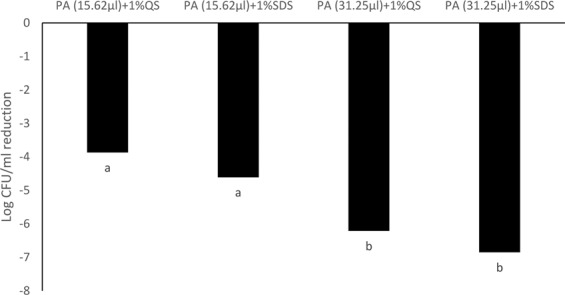
Antimicrobial efficacy of PA-SDS (1%) and PA-Quillaja saponin (1%) emulsions at concentrations of 31.25 and 15.62 mM against a *Salmonella* cocktail consisting of *S*. Newport, *S*. Typhimurium and *S*. Oranienburg after 30 s. Treatments resulting reductions of *Salmonella* cocktail that are significantly different (p < 0.5) have been indicated by different alphabet.

After 5 min of exposure no significant differences were observed in antimicrobial activity between PA emulsions generated using 1% SDS (6.85 log CFU/ml) or 1% Quillaja saponin (6.21 log CFU/ml) when used at a concentration of 31.25 mM (Fig. 9). The antimicrobial efficacy of PA emulsions tested at 31.25 mM was significantly higher than PA emulsions tested at 15.26 mM (p < 0.05) (Fig. 9). PA (15.26 mM) emulsions generated using 1% SDS or Quillaja saponin resulted in 4.61 and 3.87 log CFU/ml reduction of *Salmonella* population in the cocktail. PA concentration and duration of exposure significantly influenced antimicrobial activity of the emulsions (p < 0.05) (Fig. 9).

## Discussion

PA is a fatty acid commonly found in plants such as tomatoes and is an FDA approved food additive^26^. PA was found to have inhibitory activity against *Salmonella* at a concentration of 125 mM when dispersed in water by physical agitation^11^. When emulsified using Quillaja saponin, the inhibitory activity of PA significantly increased^11^. Hence in the current study, emulsions of PA were formed using a variety of surfactants at different concentrations and evaluated for their inhibitory activity against *Salmonella* Serotypes Newport, Typhimurium and Oranienburg. Of the surfactants tested in the study Quillaja saponin, SDS and Tween 80 are approved by the FDA as GRAS and TritonX 100 is approved for the dispersal of pesticides and applications associated with food packaging^27^.

All the surfactants used in the current study improved the inhibitory activity of PA against *Salmonella* in comparison to the control (125 mM) (Fig. 1). The antimicrobial properties of a micelle depends on its availability in the aqueous phase^28^. Emulsions of essential oils generated using surfactants such as Tween 80 or Triton X100, which are non-ionic in nature, had lower antimicrobial efficacy against *Salmonella* Enteritidis, *Escherichia coli* O157:H7, and *Listeria monocytogenes* than the unmodified essential oils^28–30^. The current study determined that PA emulsions formed using Tween 80 and Triton X100 had lower (p < 0.05) inhibitory activity against all three *Salmonella* serotypes, in comparison to Quillaja saponin and SDS (Fig. 1). It was observed that surfactant type and concentration significantly affected both, inhibitory and bacteridical activities of PA (p < 0.05). The concentration of surfactant at which its molecules aggregate to form micelles is known as the critical micelle point (CMC)^31,32^. The CMC of SDS is 2.1 g/l, Tween 80 is 0.013 g/l, Triton X100 is 0.15 g/l and Quillaja saponin is 0.8 g/l^29,31,33,34^. Surfactants used at 1% were all above the CMC and could have contributed to the antimicrobial activity of micelles. Tween 80, Triton X100 and Quillaja saponin exceeded the CMC at 0.1% and Tween 80 was concordant to the CMC at 0.01%.

Among the surfactants tested, SDS based emulsions had the largest PA micelles, as well as a high level of variation in the size of the colloidal particles generated (as denoted by the polydispersity index) compared to emulsions generated using other surfactants.

*S*. Newport was generally more sensitive to PA emulsions than serotypes Typhimurium and Oranienburg (p < 0.05). Differences in the sensitivity of *Salmonella* serotypes to antimicrobial compounds can occur and can result in higher incidences of specific serotypes in certain food commodities^35^. For instance, *S*. Oranienburg has been isolated from dry spices and has a higher tolerance to natual antimicrobial compounds in spices^36^. Pelargonic acid exposure to bacterial cells at lethal concentrations results in cell membrane damage and lysis^11^, similar to bacterial cells exposed to carvacrol, the active component of Oregano oil^11,37^. Analysis of growth curves of the *Salmonella* serotypes exposed to MIC of PA emulsions generated using 1% SDS and Quillaja saponin respectively, resulted in a reduction in growth rate and an increase in lag phase duration (Fig. 3). The formation of bacterial filaments, which are elongated structures containing many cells that remain attached due to a block in the divisome, could contribute to the reduction in growth rate as filaments are detected as a single colony during plating on bacteriological media^19,38^.

Microscopy indicated that while cells of all three serotypes formed filaments when exposed to PA emulsions, the ratios of filaments to regular cells were different (Table 2). Further, *S*. Typhimurium and *S*. Newport cells formed free-floating aggregates of floccular biofilms, whereas *S*. Oranienburg did not, indicating that stress response to PA varied among *Salmonella* serotypes. Changes in bacterial morphology among *Salmonella* serotypes occurred both during the use of Quillaja saponin and SDS as surfactants for the formation of PA emulsions. *S*. Oranienburg cells formed filaments that were longer than 5 µm and several filaments exceeded 100 µm in length (Fig. 4). Filaments are transient phenotypes that contain chains of cells connected one to another due to incomplete cell division^20^. Filaments disintegrate when cell division is completed and result in spontaneous release of many individual cells^19,20^. *S*. Typhimurium and *S*. Newport also formed filaments, but they occurred less frequently and were usually shorter (30≥µm) than *S*. Oranienburg filaments (100≥µm), (Table 2, Figs. 4, 7). Apart from filaments, *S*. Typhimurium and less frequently *S*. Newport formed aggregates that were encased in EPS (Figs. 5, 6). Autoaggregation of cells is an important precursor to biofilm formation^39,40^. The free-floating aggregates of *S*. Typhimurium and *S*. Newport were encased in a dense polymeric matrix that was observed through fluorescent staining and the use of LSCM (Figs. 5, 6). The formation of floccular biofilms as well as filamentation suggest another, a less explored bacterial strategy to mitigate antimicrobial exposure (Fig. 6)^40^. Filaments formed by *S*. Newport, the serotype most susceptible to PA, often appeared to be disfigured indicating possible structural damage to the cells (Fig. 3). All three serotypes displayed phenotypic diversity by forming filaments (Figs. 4, 5, 7), floccular biofilms and regular size cells when exposed to PA indicating that the formation of diverse phenotypes might be a strategy employed by *Salmonella* to overcome stressors such as sanitizer exposure^38^.

Emulsions have many applications in the food industry and can be used in antimicrobial formulations and coatings^41–44^. Antimicrobial emulsions can improve the safety of foods, feeds and food contact surfaces. PA emulsions (31.2 mM) generated using 1% SDS resulted in a 4.7 log CFU/ml reduction of the *Salmonella* cockatail containing all three serotypes in 30 s and 6.8 log CFU/ml in 5 min (Figs. 8, 9). A similar reduction of *Salmonella* serotypes was observed using a PA emulsion generated using Quillaja saponin after 5 min but not at 30 s, indicating that the type of surfactant selected might also effect the time taken for the interaction between the fatty acid and the target pathogen.

## Conclusions

The current study demonstates the antimicrobial activity of PA against *Salmonella* and for the first time reveals that surfactant type plays an important role in influencing the antimicrobial efficacy of PA emulsions. Further, the observation that *Salmonella* employs multiple stress responses among a clonal population of cells when challenged with an antimicrobial, indicates that phenotypic diversity may play an important role in antimicrobial resistance. This new information will help industry better evaluate their choices of antimicrobials, as inhibitory concentrations of certain sanitizers could vary greatly from bactericidal concentrations, depending on the type of surfactant and surfactant concentration chosen. The finding that the use of surfactants can reduce the amount of PA required for inhibitory activity against *Salmonella* by 50% or greater, could help in reducing the environmental footprint, the cost associated with sanitizer production and the development of alternatives to biocides currently used by the food industry.
